# Sulfotransferase and Heparanase: Remodeling Engines in Promoting Virus Infection and Disease Development

**DOI:** 10.3389/fphar.2018.01315

**Published:** 2018-11-22

**Authors:** Dominik D. Kaltenbach, Dinesh Jaishankar, Meng Hao, Jacob C. Beer, Michael V. Volin, Umesh R. Desai, Vaibhav Tiwari

**Affiliations:** ^1^Department of Biomedical Sciences, College of Graduate Studies, Midwestern University, Downers Grove, IL, United States; ^2^Department of Ophthalmology & Visual Sciences, University of Illinois at Chicago, Chicago, IL, United States; ^3^Chicago College of Pharmacy, Midwestern University, Downers Grove, IL, United States; ^4^Chicago College of Osteopathic Medicine, Midwestern University, Downers Grove, IL, United States; ^5^Department of Microbiology & Immunology, College of Graduate Studies, Midwestern University, Downers Grove, IL, United States; ^6^Department of Medicinal Chemistry and Institute for Structural Biology, Drug Discovery and Development, Virginia Commonwealth University, Richmond, VA, United States

**Keywords:** heparan sulfate, herpes simplex virus, heparanse, sulfotranferases, heparan mimetic, viral entry

## Abstract

An extraordinary binding site generated in heparan sulfate (HS) structures, during its biosynthesis, provides a unique opportunity to interact with multiple protein ligands including viral proteins, and therefore adds tremendous value to this master molecule. An example of such a moiety is the sulfation at the C3 position of glucosamine residues in HS chain via 3-*O* sulfotransferase (3-*O*ST) enzymes, which generates a unique virus-cell fusion receptor during herpes simplex virus (HSV) entry and spread. Emerging evidence now suggests that the unique patterns in HS sulfation assist multiple viruses in invading host cells at various steps of their life cycles. In addition, sulfated-HS structures are known to assist in invading host defense mechanisms and initiating multiple inflammatory processes; a critical event in the disease development. All these processes are detrimental for the host and therefore raise the question of how HS-sulfation is regulated. Epigenetic modulations have been shown to be implicated in these reactions during HSV infection as well as in HS modifying enzyme sulfotransferases, and therefore pose a critical component in answering it. Interestingly, heparanase (HPSE) activity is shown to be upregulated during virus infection and multiple other diseases assisting in virus replication to promote cell and tissue damage. These phenomena suggest that sulfotransferases and HPSE serve as key players in extracellular matrix remodeling and possibly generating unique signatures in a given disease. Therefore, identifying the epigenetic regulation of *O*ST genes, and HPSE resulting in altered yet specific sulfation patterns in HS chain during virus infection, will be a significant a step toward developing potential diagnostic markers and designing novel therapies.

## Introduction

Heparan sulfate (HS) is a linear polysaccharide ubiquitously present in all tissues. HS is attached to the cell surface or extracellular matrix proteins where it exists as heparan sulfate proteoglycans (HSPGs). Because of its unique structural capability, HSPGs serve as “heavy duty engines” by interacting with an array of multiple and diverse protein ligands to participate in virtually any given biological reaction (Yanagishita and Hascall, 1992; Sasisekharan and Venkataraman, 2000; Esko and Lindahl, 2001; Varki, 2002; Bishop et al., 2007). HS undergoes numerous modifications during its biosynthesis. One family of enzymes called sulfotransferases, which catalyzes sulfonation specific sites on HS and are recognized to play various important roles ranging from impacting cellular processes to microbial pathogenesis and associated inflammations. Infectious disease literature documents two-types of HS for their contributions in viral infections. In the first category, the plain-type or unmodified form of HS contains specific types or arrangements of sulfated residues, which assists multiple viruses for cell attachment or binding (WuDunn and Spear, 1989; Feldman et al., 2000; Shukla and Spear, 2001; Jiang et al., 2012; Richards et al., 2013; Tan et al., 2013). This is considered the first step toward establishing a successful infection. In the second category, an exceedingly specialized form of HS containing unique patterns of sulfation generated by sulfotransferase, is utilized by viruses to further facilitate infectious processes like virus-cell fusion, internalization, and trafficking etc (Shukla et al., 1999; Zautner et al., 2006; Borst et al., 2013; Connell and Lortat-Jacob, 2013; Makkonen et al., 2013).

## Synthesis of heparan sulfate (HS)

**Figure 1:**
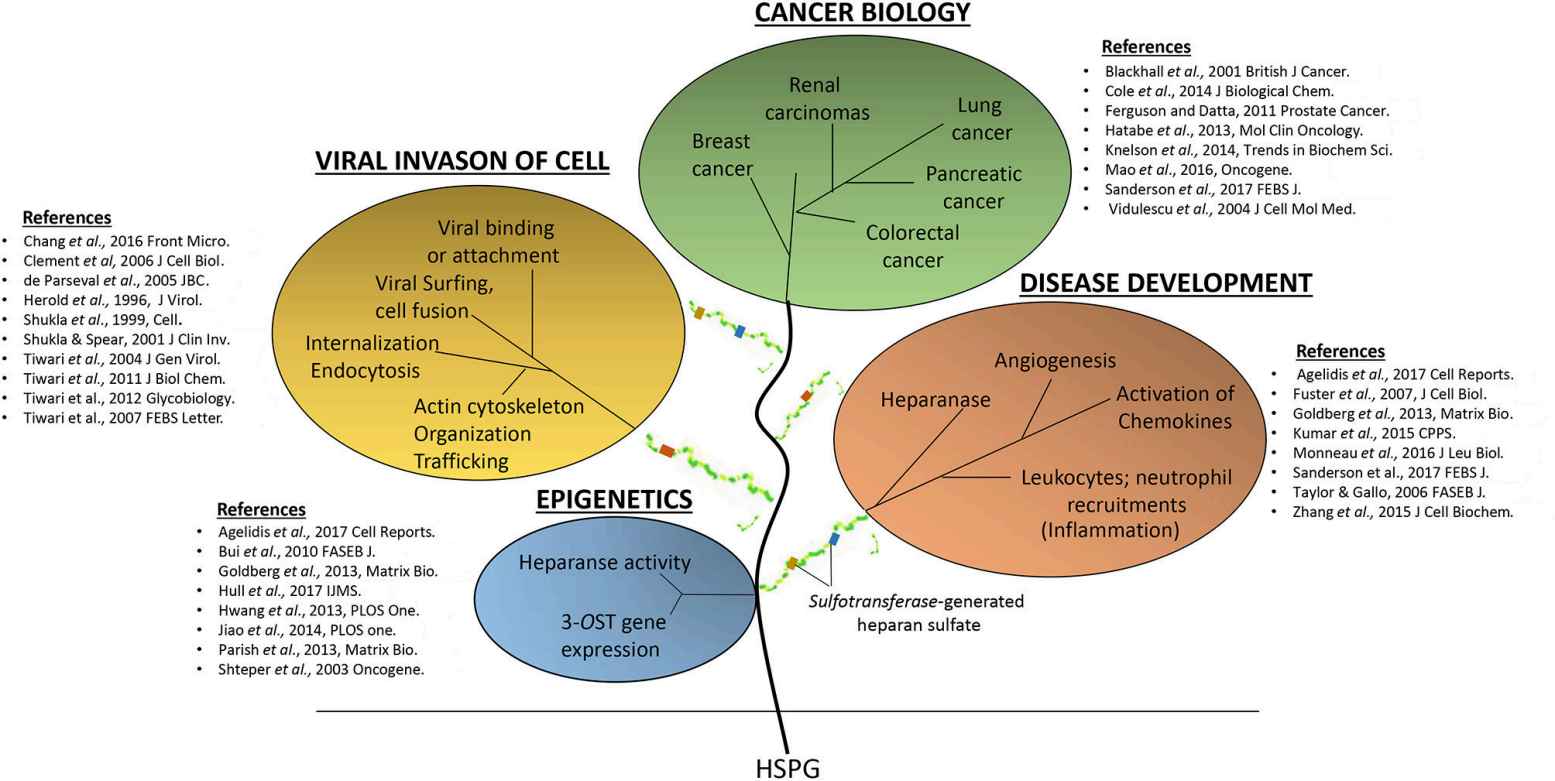
The detrimental effects of heparan sulfate (HS) and modified-forms of HS in various pathological events ranging from supporting virus invasions into host cell, multiple cancers, and disease development.

HS synthesis is a dynamic process which initially begins with addition of tetrasaccharide linker region (GlcA-Gal-Gal- Xyl) on serine residues of the syndecans- the protein core (Esko and Lindahl, 2001). Following the initial addition of an *N*-acetylated GlcN (GlcNAc) residue to the beginning of the HS chain, polymerization continues with the addition of alternating GlcA and GlcNAc residues. The polymeric chain extension is also accompanied by a series of modifications in which multiple enzymes participate in a sequential order. These include glycosyltransferases, an epimerase, and sulfotransferases (de Agostini et al., 2008). During the initial process, N-deacetylation and N-sulfation of GlcNAc occur, transforming the glycosaminoglycan into *N*-sulfo-GlcN (GlcNS). Next C5 of GlcA is epimerized to IdoA, followed by *O*-sulfation, which is performed by 2-*O*-sulfotransferases (2-*O*STs), 6-*O*ST or 3-*O*ST in the following order: Initially, 2-*O*-sulfation of IdoA and GlcA occurs followed by 6-*O*-sulfation of GlcNAc and GlcNS units, and lastly 3-*O*-sulfation of GlcN residues (Esko and Lindahl, 2001). The final step of 3-*O* sulfation is a rare modification that involves a finite number of chains (Marcum et al., 1986; Zhang et al., 2001; Thacker et al., 2014, 2016). Various arrangements of these modified residues result in a heterogeneous structure of HS which creates distinct binding motifs on the HS chains which are thought to regulate its functional specificity in distinct biological processes within the host. Concurrently, modifications of HS residues allow for distinct functions in pathogen–host interactions highlighting the impact of HS in disease development. For instance, the importance of HS-signaling in cancer biology, tumor development, metastasis, and differentiation are emerging (Figure 1) (Blackhall et al., 2001; Raman and Kuberan, 2010). Furthermore, a vast spectrum of pathogens ranging from viruses, bacteria, parasites, and fungi exploit the various moieties present on HS to signal and facilitate microbial pathogenesis (Sinnis et al., 2007; Gardner et al., 2011; Tiwari et al., 2012; Park and Shukla, 2013; Jinno and Park, 2015; Lin et al., 2015; Raman et al., 2016). HS promotes both, initial microbial attachment and associated inflammatory response, which may result in damaging outcomes for the host (Urbinati et al., 2009; Xu et al., 2011; Axelsson et al., 2012; Knelson et al., 2014; Yun et al., 2014; Kumar et al., 2015; Dyer et al., 2016).

## Sulfotransferase generated heparan sulfate

**Figure 2:**
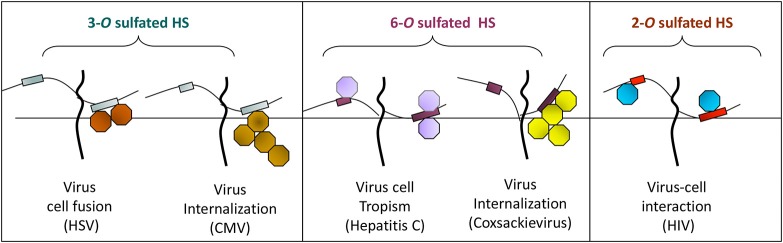
The *O*-sulfation in HS chain is known to generate binding sites for multiple viral proteins to facilitate viral entry. For instance, sulfation at 3-*O* position on glucosamine residues generates a HSV-1 glycoprotein D (gD) receptor for entry. In addition, 3-*O* sulfation in HS facilitates the spread of cytomegalovirus (CMV). Similarly, 6-*O* sulfation in HS chain promotes hepatitis C and coxackievirus entry. Interestingly, it has been demonstrated that HIV glycoprotein gp120 interacts with 2-*O* sulfated HS during cell entry.

Currently only three sulfotransferases: 2-*O*, 3-*O*, and 6-*O* sulfotransferase (2-*O*ST, 3-*O*ST, and 6-*O*ST) enzymes are known to generate 2-*O*, 3-*O*, and 6-*O* sulfated heparan sulfate (2-*O*S HS, 3-*O*S HS, and 6-*O*S HS) respectively. These sulfated forms of HS play a role by generating high affinity receptors that allow viral and bacterial pathogens to have affinity for their natural target cell-types (Clement et al., 2006; Tiwari et al., 2006, 2007, 2011; Zautner et al., 2006; Trottein et al., 2009; Kobayashi et al., 2012; Hayashida et al., 2015; García et al., 2016) (Table 1 and Figure 2). Gene profiles for sulfotransferases have shown an enhanced expression in human monocytes and dendritic cells (DCs) during differentiation suggesting their role in responding to pathogens, stress, and during aging (Taylor and Gallo, 2006; Sikora et al., 2016). Since a large array of inflammatory and immunoregulatory mediators are known to interact with cell surface HS to target subsets of T lymphocytes and monocytes/macrophages (Zhou et al., 2015), dissecting such interactions could generate a valuable tool useful for understanding inflammatory and immune responses. Moreover, the impact of specific ligands on sulfotransferase enzymes and its turnover during pathogen invasion requires additional detailed investigation. In fact, a recent study offers oligosaccharides as a means by which to inhibit HS- sulfotransferases, adding new tools to probe the structural selectivity for HS-binding proteins (Raman et al., 2013).

**Table 1:** *O*-Sulfation in heparan sulfate and its implication in pathology.

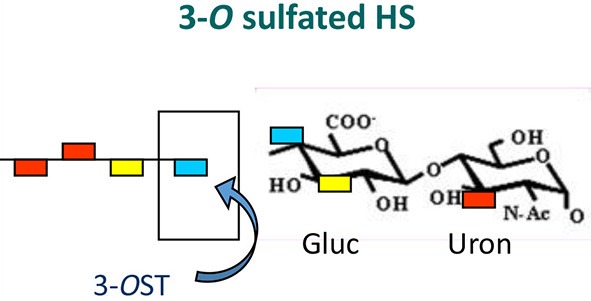	3-*O*ST-1 3-*O*ST-1,2 3-*O*ST-1,5 3-*O*ST-2 3-*O*ST-1-7 3-*O*ST-3B 3-*O*ST-3B 3-*O*ST-3B1 3-*O*ST-3B1 3-*O*ST-7	Insulin secretion (Takahashi et al., 2012). Axonal growth; endothelial sprouting (Zhang et al., 2015a). Antithrombin binding (Mochizuki et al., 2003). Hypermethylation as marker in multiple cancers (Miyamoto et al., 2003). Zebrafish development (Cadwallander and Yost, 2006). Inflammatory stimuli in Monocytes (Sikora et al., 2016). Promotes Angiogenesis (Zhang et al., 2001). Marker for breast cancer (Mao et al., 2016). Novel inducer of Pancreatic cancer (Song et al., 2011). Cardiac Rhythm (Samson et al., 2013).
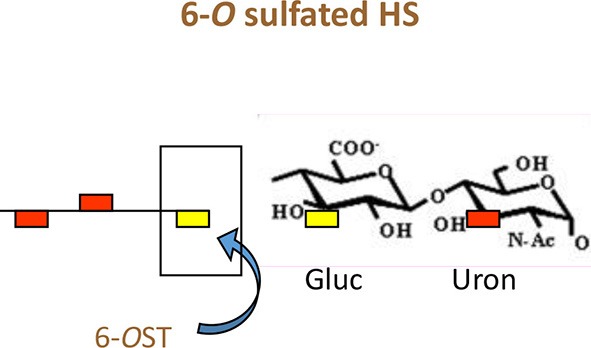	6-*O*ST 6-*O*ST 6-*O*ST 6-*O*ST 6-*O*ST 6-*O*ST-1 6-*O*ST-2 6-*O*ST-2 6-*O*ST-3 6-*O*ST-3	Angiogenic programming (Ferreras et al., 2012). Vascular development (Chen et al., 2005). Organogenesis (Sedita et al., 2004). Muscle development (Bink et al., 2003). Chronic renal fibrosis (Alhasan et al., 2014). Adrenaline stimulation (Nishida et al., 2014). Regulating chondrocyte growth (Wang et al., 2015). Colorectal cancer (Hatabe et al., 2013). Bone marrow differentiation (Zhao et al., 2015). Gene polymorphism with Obesity (Wang et al., 2013).
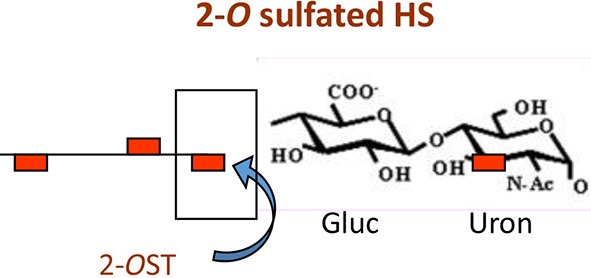	2-*O*ST 2-*O*ST 2-*O*ST 2-*O*ST 2-*O*ST 2-*O*ST 2-*O*ST	Neuron migration/axon guidance (Tillo et al., 2016). Modulating actin cytoskeleton (Zhang et al., 2015b). Antibacterial Innate immunity (Xu et al., 2015). Prostrate cancer (Ferguson and Datta, 2011). Retinal axon guidance (Irie et al., 2002). Cell migration (Kinnunen et al., 2005). Lipoprotein clearance (Stanford et al., 2010).

Interestingly, these modified versions of HS are also increasingly recognized as potential markers for invasive diseases such as cancers and tumors. It has been proposed that 3-*O*S HS and 6-*O*S HS reinforce cancer cells to break down the extracellular matrix (ECM) to spread and highjack normal signaling pathways to facilitate cell spread (Brennan et al., 2016). In addition, HS structures containing unique subsets of sulfated domains are also proving to be facilitators for inflammatory responses making them specialized HS—a high-value molecule to be exploited for future drug development. In fact, engineered structure-specific HS-analogs or HS glycomimetics designed to modulate a specific function, such as to enhance protein interaction or to modulate the inflammatory process for overall higher efficacy and lower toxicity, are now routinely synthesized as promising new drugs for use against cancer and multiple other diseases (Esko and Selleck, 2002; Zhou et al., 2015; Afratis et al., 2017; Sanderson et al., 2017).

The unique chain that results from modifying HS enables it to bind specifically to its ligand to modulate angiogenesis, axonal sprouting, and insulin secretion (Liu and Thorp, 2002; Takahashi et al., 2012; van Wijk and van Kuppevelt, 2014; Zhang et al., 2015a). The many and diverse effects of the use of these drugs can results in better control of many different disease processes with minimal side effects and ultimately bring about better patient outcomes.

### Sulfotransferase generated heparan sulfate in HSV entry

Modification also permits viral membrane fusion and penetration (Shukla et al., 1999; Xia et al., 2002; Chen et al., 2008). Unique sites within HS chains generated by 3-*O*STs produce 3-*O*S HS, the newest family of HSV-1 glycoprotein D (gD) receptors, (Liu et al., 2002; Borst et al., 2013). The 3-*O*STs act to modify HS further along in its biosynthesis, and each isoform recognizes glucosamine residues, as substrates, within regions of the HS chain having specific and unique, prior modifications, including epimerization and sulfation at other positions (Liu et al., 1999; Shworak et al., 1999). Thus, each 3-*O*ST is capable of producing potentially unique protein-binding sites within HS. To date, seven different isoforms of human 3-*O*STs (3-*O*ST-1, 3-*O*ST-2, 3-*O*ST-3 A, 3- *O*ST-3 B, 3-*O*ST-4, 3-*O*ST-5, 3-*O*ST-6, and 3*O*ST-7) are known. Although all the isoforms modify HS to generate 3-*O*S HS, it remains a mystery as to why seven isoforms are present. All, forms of 3-*O*ST except 3-*O*ST-1, produce HSV-1 entry receptors (Liu et al., 1999; Shukla et al., 1999). Interestingly, zebrafish embryo encodes 3-*O*ST isoforms that are functionally similar in terms of recognizing HSV-1 glycoprotein D (gD) to promote viral entry. The gD receptors generated by other isoforms of 3-*O*ST are very similar in structure, but likely not identical (Liu et al., 1999; Shukla et al., 1999; Chen et al., 2008; Borst et al., 2013). The isoform 3-*O*ST-1 creates binding sites for antithrombin (Shworak et al., 1999; Mochizuki et al., 2003) but are unable to produce receptors for HSV-1 gD (Borst et al., 2013). Additionally, one or more isoforms of 3-*O*STs have been found to be expressed in both human and mouse tissues that are relevant to HSV-1 infection examined thus far (Liu et al., 1999; Mochizuki et al., 2003; Lawrence et al., 2007) suggesting the significant role 3-*O*ST play in contributing to disease development. The general distinction between plain-type of HS and the HS chain containing 3-*O* sulfation regarding their usage in viral entry is well-documented. Although the vast jungle of HS in the host cell assists in the capture and concentration of pathogens at a given site, it may not guarantee successful infection since HS mainly serves as an attachment receptor. Overall interactions between HS and pathogens are considered to be non-specific and may influence pathogenesis indirectly. On the other hand, target cells expressing 3-*O* or 6-*O* sulfated moieties in HS chains may guarantee that certain pathogens will be able to cross the host cell membrane and therefore the presence of the later form of specialized-HS may serve as a “Wi-Fi” zone for the targeting pathogens to easily invade the host cell. That is, pathogens in the vicinity of cells expressing modified HS can connect to “the internet” (host organism) by using modified HS as a means and signal to literally connect to host cells. To further the analogy, pathogens may even be able to detect modified HSPGs similar to how one can scan for Wi-Fi signals with a computer or a cellphone. Nonetheless, it remains to be investigated whether such “Wi-Fi” zones can be distinctly equipped with a particular signature of the 3-*O*-sulfated isoform that influences or contributes to establishing HSV tropism. Moreover, a HSV-1 gD-type 3-*O* HS specific to neurons or to ocular cells are well-documented (Clement et al., 2006; Deligny et al., 2010).

**Figure 3:**
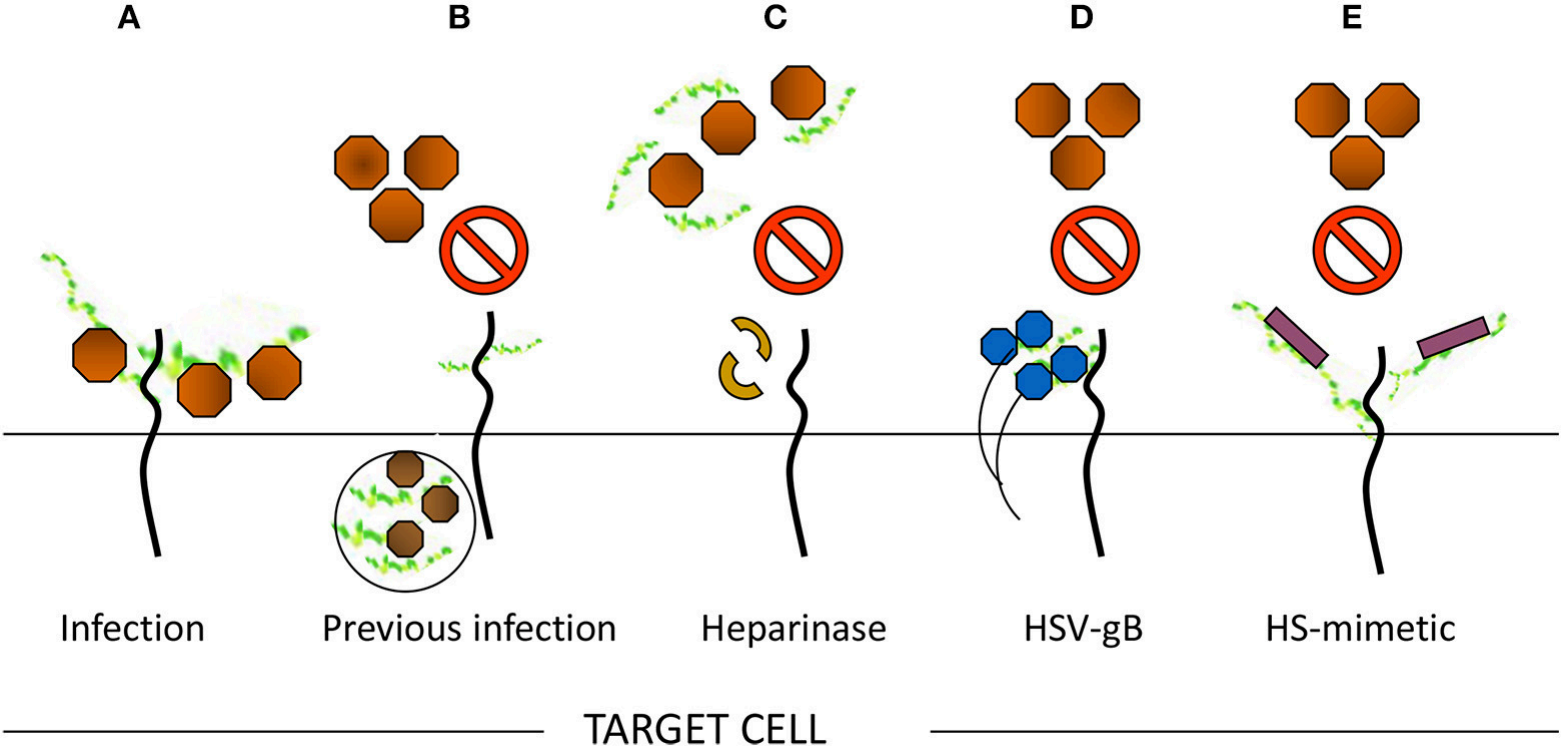
Multiple ways to demonstrate the significance of heparan sulfate (HS) in viral entry. **(A)** Moieties of HS chain provide initial docking sites for viral attachment, while sulfated HS chains generate receptors to promote virus-cell fusion. In contrast, the loss of HS may result in significant reduction of viral entry. For instance, **(B)** previous viral infection may result in resistance for incoming virus due to loss of HS. **(C)** In addition, enzymatic treatment of cells with heparanase may remove the critical moieties in HS chains required for viral entry and therefore results loss of viral infection. **(D)** Sequestering HS chain from the cell surface upon expressing viral protein renders resistance to in coming virus. **(E)** A competitive inhibition of viral entry by usage of HS mimetic on cell surface.

A series of evidence support the role of HS in viral infection as indicated in Figure 3. For example, enzymatic removal of specific regions in the HS chain in target cells by using heparanase significantly impairs the viral infection (Tiwari et al., 2004). Likewise, use of soluble heparin or HS-mimetic analogs inhibits viral infection through direct competition for cell surface HS (Gangji et al., 2018). Furthermore, a higher degree of viral entry was noticed in the cells that expressed HS compared to the mutant cells that were devoid of or weakly expressed HS. Lastly, the expression of viral envelop proteins which interact with HS in target cells, resulted in a resistance to viral entry by sequestering cell surface HS (Tiwari et al., 2006). As in the case of HSV-1, the expression of viral envelop glycoprotein B (gB) in target cells, which is known to bind HS, significantly impairs viral entry- a process also known as gB-mediated interference. Profoundly, our initial work made a unique discovery that viruses do in fact utilize HS beyond cell attachment and binding. Using cDNA library screening, direct evidence was presented for a modified version of HS; 3-*O*S HS generated by 3-*O*ST-3 isoform as a receptor which promotes virus-cell membrane fusion (Shukla et al., 1999). Expression of the HS-modifying 3-*O*ST-3 enzymes confers susceptibility for HSV-1 infection in previously HSV-1 resistant Chinese hamster ovary (CHO-K1) cells (Shukla et al., 1999). Additionally, an event with the protein receptors (nectin and HVEM) was observed that supports HSV-1 entry (Shukla and Spear, 2001). After identifying the significance of 3-*O*ST-3 in HSV-1 entry, our group has cloned an additional six individual human and zebrafish isoforms of 3-*O*ST enzymes and have characterized them against HSV-1 entry. Given the fact, that these enzymes are tightly regulated and are expressed in specific cells and tissues, it is tempting to speculate that 3-*O*ST isoforms might be prime candidates for HSV localized infection either in brain or ocular cells and tissues, essentially contributing toward viral tropism. In this regard, our previous work has demonstrated the significance of one 3-*O*ST-3 isoform type in primary cultures of human corneal stroma (CF) derived from eye donors- a natural target cell for HSV infection. Using si-RNA approaches together with the use of heparanase enzyme in cultured CF, we found a significant reduction in HSV-1 entry (Tiwari et al., 2006). Additional support for HSV use of the 3-*O*S HS receptor in CF was gained by using phage display-derived peptide targeting 3-*O*S HS receptor, which further resulted in a compelling inhibition in HSV-1 entry (Tiwari et al., 2011). To further strengthen our work, we used anti-3-*O*S HS peptides to successfully prevent HSV-1 infection in a mouse corneal model (Tiwari et al., 2011). With the above encouraging results, we have tested the clinical usage of anti-3-*O*S HS peptides by using them on a commercially available contact lens as a delivery vehicle for extended release as a potential and effective way to control corneal herpes infection (Jaishankar et al., 2016). Interestingly, a laboratory produced 3-*O*-sulfonated HS octasaccharide, was demonstrated to inhibit HSV-1 and host-cell interactions, suggesting the application of HS derived molecules as potential therapeutic tools against viral pathogens.

In the case of 3-*O*S HS, the ligands that are documented to interact are HSV-1 glycoprotein D (gD), antithrombin III, Fibroblast growth factor (FGF), Cyclophlin-B, and Neuropilin-1 (Liu et al., 1999; Datta et al., 2013; Baldwin et al., 2015; Zhang et al., 2015a). Emerging evidence suggests that multiple viruses have evolved with the interaction of *O*-sulfated domains in HS to promote infection. For instance, 3-*O*S HS although first recognized to be HSV-1 gD receptor, is now reported to be utilized by other members of the herpes virus family as well. A recent study by Baldwin et al. (2015) demonstrated that human cytomegalovirus interacts with 3-*O*S HS generated by 3-*O*ST-3 isoforms to mediate virus spread (Zhang et al., 2010). This result emphasizes the symbolic role of 3-*O*S HS in co-infection or in superinfection models especially since herpes viruses are opportunistic by nature. On the other hand, it is also possible that viruses keep a secret and independent life in the infected cell by down-regulating the expression of 3-*O*S HS to make supportive habitats in the infected cells for their own distinct advantage. This question remains unresolved and needs to be further addressed. Similarly, 3-*O*S HS is exploited by viruses as an entry receptor, but at the line of duty, it primarily exists as a signaling transduction molecule. Therefore, it is possible that viruses intelligently sense the 3-*O*S HS environment and as such, they have no control over their turnover. Clearly, all the above questions are extremely vital as HS/3-*O*S HS widely participates in various steps of the virus life cycle.

### Role of sulfotransferase generated heparan sulfate in other viruses

Interestingly among non-herpes viruses, expression of human 3-*O*ST-3a may suppress Hepatitis B virus replication in hepatocytes (Hallak et al., 2000), while 6-*O* sulfation in HS chain potentially supports entry of human cytomegalovirus (Zautner et al., 2006). In addition, 6-*O*S HS has been shown to mediate internalization of coxackievirus B3 (Connell and Lortat-Jacob, 2013). Interestingly, 2-*O* sulfation is recognized by HIV glycoprotein gp120 during cell entry (Figure 2) (Makkonen et al., 2013; Matos et al., 2014). Conversely, N-sulfation of heparan is necessary for inhibition of Respiratory Syncytial Virus (RSV) infection (Fechtner et al., 2013) and is required for CHIKV infection (Tanaka et al., 2017). In case of Hepatitis C Virus (HCV), envelope glycoprotein E1/E2 requires both N and 6-*O* sulfate group to interact with HS (Trottein et al., 2009).

Besides viruses, other pathogens can also interact with the modified product of HS. For instance, an outer membrane protein of *Chlamydia trachomatis* known as OmcB interacts with 6-*O*S HS during infection (Tanaka et al., 2012). Seemingly, HS/3-*O*S HS becomes an ideal target for both viral and bacterial infection- a possibility that exists in many sexually transmitted infections. In the case of human T-cell leukemia virus type 1 (HTLV), it has been demonstrated that the combination of the number and the length of HS chains containing heparin-like regions is a critical factor in determining cell tropism of HTLV-1 (Monneau et al., 2016). It is hypothesized that shorter HS chains may be able to induce receptor complexes (HTLV-1 Env-HS-NRP-1-GLUT1) more efficiently than their longer counterparts by attracting HTLV-1 particles to the target cell surface. Nonetheless, the findings obtained in this study may provide the foundation for the development of effective therapies against HTLV-I infection and aid in the development of a metric for assessing the prognosis of HTLV-1 induced diseases. Whether the length of the HS chain influences virus infection in general still requires further investigation. Furthermore, whether the virus infection subverts the length of HS or effects HS-polymerization is also unrecognized. However, recent progress made in glycobiology has shown that glycosaminoglycan structural properties such as length, sulfation, and epimerization patterns, are cell, tissue, and developmental stage specific (Patel et al., 1993).

It is clear that a single type of sulfation in a HS chain can serve as an attachment point for multiple types of viruses. For instance, 3-*O* sulfation in HS generated by 3-*O*ST-3 is utilized by both HSV and cytomegalovirus (CMV) to trigger virus entry. Moreover, an individual virus can exhibit the preference to two entirely different sulfation patterns in HS chains (Shukla et al., 1999; Baldwin et al., 2015). According to recent studies, CMV can facilitate both 6-*O*S HS and 3-*O*S HS for infectious entry (Borst et al., 2013; Baldwin et al., 2015). Though it is still unknown for many viruses, what specific sulfonation pattern is most preferred.

An exponential amount of evidence suggests that HSPGs play a significant role in the HIV lifecycle and disease development. Initially, it was demonstrated that viruses enter multiple T cells by using HS chains of HSPGs to facilitate entry (Roderiquez et al., 1995). Concomitantly, the presence of heparanase has been shown to competitively inhibit HIV entry into the target cells (Roderiquez et al., 1995; Ohshiro et al., 1996). In addition, multiple studies demonstrated that the presence of HSPG in various cell types enhances HIV entry (Ibrahim et al., 1999; Saphire et al., 2001; Guibinga et al., 2002; Zhang et al., 2002; Bobardt et al., 2004). For instance, primary human endothelial cells which are loaded with HSPG are extremely efficient in capturing viruses on the cell surface (Argyris et al., 2003). The overexpression of HS in primary endothelial cells near the blood-brain barrier and in the microvasculature, in addition to their ability to capture HIV-1, has been proposed as a novel mechanism to facilitate the invasion of the brain by HIV-1 (Argyris et al., 2003). Further prime target cells like DCs, macrophages, epithelial, and endothelial cells provide docking sites for HIV by expressing HS (Guibinga et al., 2002; Wu et al., 2003; Gallay, 2004; de Witte et al., 2007; Ceballos et al., 2009).

Additionally, HS-expressing spermatozoa play a crucial role in virus transformation into DCs, macrophage and T cells during HIV transmissions (de Parseval et al., 2005). In fact, four HS-binding domains have been identified in the V2 and V3 loops, the C-terminal domain, and the CD4-induced bridging sheet of the HIV gp120 (Crublet et al., 2008; Herrera et al., 2016). In contrast, one recent study depicts that HS combines with the innate protein human beta-defensin (hBD) and reduces HIV trans-epithelial transmission through inactivation of virus. Using an adult oral epithelial cell which expresses hBD, the authors showed the HSPG-mediated internalization of hBD and HIV-gp 120 in the endosome results in oligomerization and reduced infectivity of HIV (Vivès et al., 2005).

Remarkably, it has been demonstrated that HS not only facilitates viral attachment to host cells via HIV gp120 (Cladera et al., 2001) but also that HS mediates viral-host fusion by interacting with the fusion domain of gp41 (Teixé et al., 2008). On permissive cells, HIV binding to HS is thought to increase infectivity by concentrating viral particles at the cell surface (in the *cis* process). Additionally, for some cell types such as macrophages, HS may compensate for low CD4 expression (Guibinga et al., 2002). It has also been proven that the expression of HSPG on the CD4+ cell surface is regulated by virus infections and immune activation (Ibrahim et al., 1999; Alvarez Losada et al., 2002). HS may be directly involved in the infection of CD4^+^ and/or CD4^−^ cells, making it an ideal target for new anti-HIV therapies (Wu et al., 2003; Somiya et al., 2016). Beside HS interaction to gp120, it also co-localizes with matrix protein p17 on activated human CD4^+^ T cells. Additionally, single particle tracking confirms that multivalent Tat protein transduction results in a domain-induced heparan sulfate proteoglycan cross-linkage, which activates Rac1 for virus internalization. HIV-1 Tat and HSPG interaction have been proposed as a novel mechanism of lymphocyte adhesion and migration across the endothelium (Xu et al., 2011).

Besides the symbolic role of HS for viral entry, HS plays other extensively documented roles in the viral lifecycle. Using a recombinant Hepatitis B virus (HBV) surface antigen L protein particles (bio-nanocapsules, BNCs), a HS-dependent mechanism of HBV uptake has been proposed (Dong et al., 2013). To study the hepatitis C virus (HCV), a gene silencing approach was used against enzymes involved in HS-biosynthesis to investigate structural determinants required by HCV during infection. The study found the N- and 6-*O*-sulfation, but not 2-*O*-sulfation, is required for HCV infection. It is also known that the minimum HS oligosaccharide length required for HCV infection is a decasaccharide. Overall, previous discoveries highlighted that HCV uses specific HS structure to initiate infectious cycle. Concurrently, another study implemented a different approach by using heparanase I, II, and III enzymes to cleave specific structures in HS to reach the same conclusion. The findings indicate that the heterogeneity of HS especially for rich N-sulfation and iduronic acids heavily participate in Respiratory Syncytial virus (RSV) infection in some mammalian cells (Cooper et al., 2005).

Besides the role of HS in viral entry, it is justified that HS is also involved in the induction of cytokines by HBV capsid in macrophages via TLR2 signaling (Tsidulko et al., 2015). A recent study has proposed that within various bovine tissues used for HS preparation, only liver-based HS can strongly bind to both E1 and E2 of Hepatitis C Virus (HCV). This study is very significant since HCV, which displays liver tropism, contains highly sulfated structures compared to HS from other tissues. This concludes that HS-proteoglycan on liver cell surfaces appear to be one of the molecules that define liver-specific tissue tropism of HCV (Trottein et al., 2009). Later, a study by Lawrence et al. (2007), Deligny et al. (2010) supports the notion that HS structures vary depending on the cell and tissue, and this can influence virus tropism. For instance, HS-modifying enzymes 3-*O*ST-2 and 3-*O*ST-4 are the predominant forms expressed in the neurons of the trigeminal ganglions while 3-*O*ST-2 and 3-*O*ST-4 generated HS have been shown to be of the principal gD-receptor type for HSV-1. Ultimately, all of these subtleties reinforce the presence of a selective interaction between HS and viruses. Although multiple viruses use HS proteoglycans which are ubiquitously expressed GAGs for binding and or attachment, it remains to be ruled out if virus interactions with HS are random or if they require structural specificity.

A unique pattern of HS expression in Epstein Barr Virus (EBV) infected cells is established. Lymphoblastoid cell lines infected with EBV have shown specific proteoglycan expression with down-regulation of CD44 and ECM components and up-regulation of serglycin and perlecan/HSPG2. Nevertheless, in Burkitt's lymphoma cells (BL), serglycin was shown to be down-regulated. Fundamentally, the biosynthetic machinery for HS construction and modification was active in all cell lines, with the cavate that this machinery was down-regulated in BL cells (Shannon-Lowe and Rowe, 2011). A separate study documents the involvement of sulfated-HS during B cell/epithelial cell interactions during Epstein—Barr virus (EBV) transfer (Woodard et al., 2016). In fact, understanding HS-virus association has been investigated for many, entirely different reasons to directly help patients. In ocular gene therapy, a novel chimeric adenovirus-associated vector (AAV) has been developed, which binds to HS and hence displays better transduction ability. This result has generated AAV with higher accumulation and penetration to the retina and therefore offers a less invasive intravitreal injection route for ocular gene therapy (Kaever et al., 2016). Similarly, the significance of HS in developing antibodies against vaccinia virus A27, human papillomavirus (HPV) has been advocated (Silva et al., 2014; Xia et al., 2016).

In addition, a study tested two different strains (vaccine strain and clinical isolates) of Chikungunya virus (CHIKV) for GAG binding generated efficient virus replication. Although GAGs are necessary for efficient binding of both strains, they exhibit differential requirements for GAGs. However, the study delineated the critical amino acid residue 82 in the envelope glycoprotein E2 is a primary determinant of GAG utilization. A critical outcome for the future development of viral entry inhibitors against CHIKV infection (Richards et al., 2013). Additionally, the importance of HS structure for CHIKV infection was demonstrated in a study by Tanaka et al. (2017), Following genome screening the CRISPR/Cas9 system was deployed to create knockout models of haploid human cells (HAP1). These knockout models were created to assess the importance of various genes that modify HS in efficient CHIKV infection. Infectivity was not affected following disruption of genes encoding proteins that act sequentially after NDST1, an enzyme that catalyzes the N-sulfation step of HS (Tanaka et al., 2017). GLCE is one such protein that acts after the N-sulfation step. GLCE catalyzes the C5 epimerization of GlcA to IdoA which is a necessary step required to later produce the 2 and 3-*O*-sulfated forms of HS. This phenomenon stands in contrast to the mechanism of HSV infection, where these modifications have been shown to be necessary for infection. Concerning CHIKV infection, HS in the 2 and 3-*O-*sulfated forms are not required for productive infection but rather it is the N-sulfated HS that facilitates viral binding and host cell entry.

In the case of HPV, multiple HS-binding sites were found on the capsid protein L1 which bring sequential confirmation shifts during virus-receptor engagement assisting virus attachment and internalization. For instance, primary binding is regulated by site 1 (Lys278, Lys361). It can cause confirmation shift in L2, which mediates the display of the amino terminus. In the step after primary attachment, Site 2 (Lys54, Lys356) and site 3 (Asn57, Lys59, Lys442, Lys443) are essential for viral entry (Richards et al., 2013). Overall, the HS-binding mediates exposure of secondary binding sites aiding in the virus engulfment.

There is a study comparing the amino acid sequences of the E2 glycoprotein from natural North American eastern equine encephalitis virus (NA-EEEV) isolates that shows that the neurovirulence of EEEV is supported by the interaction with HS (Voss et al., 2010). For instance, mutations from lysine to glutamine at E2 71, from lysine to threonine at E2 71, or from threonine to lysine at E2 72 were found to have an altered virulence and interaction with HS. Using an electrostatic map, the HS-binding regions in the EEEV E1/E2 heterotrimer were predicted at the apical surface of E2. Basing this method upon the recent Chikungunya virus crystal structure, variants were detected to affect the electrochemical nature of the binding site (Ryman et al., 2007). The EEEV sylvatic cycle could be the starting point for these natural variations in the EEEV HS-binding domain, which may influence receptor interaction as well as the severity of EEEV disease (Voss et al., 2010).

Similarly, HS-binding has been proposed to be an important factor in neurovirulence of neuroadapted and non-neuroadapted Sindbis Viruses (SV) (Milho et al., 2012). Substantially, HS on olfactory neuroepithelium offers an essential and multifaceted site for HS-dependent murid herpesvirus-4 uptake. Therefore, olfactory neuroepithelium poses a critical entry point for heparan-dependent herpesvirus (Antoine et al., 2014).

## Heparan sulfate- a gateway for the emerging diseases

Emerging highly pathogenic viruses continue to use cell surface HS for attachment. For instance, zoonotic viruses such as Nipah and Hendra viruses have shown to use to HS for viral entry during lethal infection (O'Hearn et al., 2015). Similarly, Filoviruses, including Ebola virus (EBOV) and Marburg virus (MARV), known to cause hemorrhagic fever with a high mortality rate are also known to use cell surface HS and enzyme EXT-1 involved in HS-biosynthesis (Riblett et al., 2016). In addition, Rift Valley fever virus (RVFV), also an emerging pathogen that can cause lethal hemorrhagic fever syndrome in humans, has been documented to use HS during the initial stages of infection (Mao et al., 2016).

## Heparan sulfate- a platform for infection

Viruses are consistently facing enormous challenges due to the jungle of HS chains on the cell surface which may hinder or prevent the virus from being released efficiently from the infected cell. Ironically this gives viruses an opportunity to get equipped with a strategy to overcome this problem. For instance, in hepatocytes hepatitis B the virus hides its envelope glycoprotein in its interior during release and becomes HSPG-non binding N-type. Then later it becomes HSPG-binding B-type followed by the translocation of its envelope protein back to the viral surface the latter of which, results in the virus's infectivity. In the case of HSV, it was recently discovered that upon infection the expression of heparanase enzyme is up-regulated via NF-kB mechanisms, which aid in cleaving off the HS/3*O*S HS chains resulting in virus release, supporting viral pathogenesis. Taken together, viruses find their way to leave the cells and prepare for next target cell by changing the landscape of their own HS-interacting envelope glycoproteins or by shedding the cell surface HS. Interestingly, an overexpression of heparanase is well-documented in multiple pathological conditions such as inflammation, angiogenesis, angiogenesis, tumor metastasis, and atherosclerosis. Again, heparanase mediated breaking or shedding of the basement membrane of epithelial and endothelial cells results in an increase in vascular permeability which in turns supports the leakiness and migration of the leukocytes in the surrounding tissues. In addition, actions of heparanase cause further damage by the release of HS-sequestered with cytokines and growth factors (e.g., vascular endothelial growth factor; VEGEF), which either on the spot or in the surrounding regions, ignite inflammatory processes and angiogenesis aggravating the issue. The later process results in the activation of GTPase signaling, which activates cytoskeleton proteins to promote cell migration and invasion. Again, these actions combined with the up-regulation of matrix metalloproteinase facilitate the intercellular tumor invasion and metastasis after the loss of the HS barrier in the ECM. Further, the released HS may bind to endogenous TLR4 which in turn-on activates the signaling cascade to release the pro-oncogenic signals. These reactions are too overwhelming for the cell and tissues to repair the damage and maintain their homeostasis. Therefore, the overall process may result in devastating disease. A similar situation exists during ocular HSV-1 infection when the virus initially mediates the infection in the layer of corneal epithelium which later brings an overwhelming immune response together with angiogenic cytokines. This immune-mediated response in the corneal stroma can compromise the corneal transparency ultimately resulting in vision loss.

The sulfated moieties present in a HS chain are key determinants for its biological significance. Analyzing HS-moieties for their structure and function is an important strategy for multiple reasons. For instance, multiple moieties in the HS structure remain “orphan” in the sense that the ligands they interact with are not yet fully discovered or if they exist, are poorly understood. In addition, the structural moieties in HS do have “good” and “ugly” components which are differentially expressed signifying a “biological clock” and further suggesting using expression pattern as a potential diagnostic tool. For example, expression of 3-*O*ST and 6-*O*ST has been shown to be up-regulated in certain cancers (Connell et al., 2012; Cole et al., 2014). Since the potential in human disease and associated pathologies including virus infection is now being recognized, identifying the vasculature of HS and associated signature of pro-inflammatory chemokines/cytokines in a diseased state is crucial for potential target development.

Interestingly, a study by Connell et al. (2012) demonstrated the concept of generating HS synthetic -mimetic peptide conjugated to a mini CD4 display high anti- HIV-1 activity independent of co-receptor usage (Whittall et al., 2013). The CD4 mimetic and the heparan sulfate derivative interact synergistically and allow potent antiviral activity against both CCR5- and CXCR4-tropic HIV-1 strains. Similarly, peptide-based molecules that directly recognize 3-*O*S HS or 6-*O*S HS or 2-*O*S HS will be of enormous value to understand multiple disease pathologies including virus pathogenesis. In this direction, we made an optimistic attempt by developing peptides which recognize HS or 3-*O*S HS. These peptides were found to be effective against a murine ocular HSV model. Further characterization of the anti-HS and anti-3-*O*S HS peptides to prevent inflammatory response need to be tested. In fact, there is a critical need to crack the code between the viruses and their affinity for specific forms of HS. We need to define their structural moieties. There is an emerging trend indicating that multiple viruses do use specific moieties present in HS chain suggesting association to the structural components. However, this remains to be determined if viruses have a preference for one type of sulfation pattern over other types. If true, it would give viruses an edge to establish tropism because cell and tissues exhibit specific expression of sulfated-HS. This could mean that viral use of a specific sulfate-HS moiety could be used as a marker for a more virulent strain. Further, is there is a unique sub-set of sulfated-HS that are expressed in disease states, which are either not present in healthy cells and tissues or present at low levels? A major gap in knowledge exists if the post virus infection influences the expression of certain types of HS-sulfotransferases or if their combinations further impact the additional variability and complexity in the existing HS structure. Developing a blueprint in the regions of the sulfated-moieties in HS during pre-and post-infection and identifying their binding partner's, especially the viral ligands, will boost the development of the targeted therapy. Our complete understanding of the structural components in HS interacting to ligands during various biological processes is very likely to advance our knowledge in the benefit of human health by preventing virus infection, serious diseases like cancer, and their associated pathologies. For instance, peptide-based formulations that directly target interactions between viruses envelop glycoprotein and respective sulfated-HS and affecting the immune response will be new exciting therapeutic options as a broad-spectrum virus inhibitor. Usage of cell surface HS moieties during viral binding and entry is a unique feature that is shared by medically important viruses including many herpes viruses. Similarly, multiple viruses exploit modified-HS differently to promote infection. For instance, HSV uses 3-*O*S HS to promote virus-cell fusion, while the presence of 3-*O*S HS significantly increases during CMV internalization, suggesting the possibility that 3-*O*S HS-mediates endocytosis (Baldwin et al., 2015). Further, use of multiple virus strains including clinical isolates and cell-types are also necessary to classify global or very specific-usage of sulfated-HS. These data will be highly informative and will allow in establishing HS-typing. One additional way is to screen the phage display library against 2-*O*, and 6-*O* sulfated-HS. Interestingly, certain structural forms of HS have been suggested to be involved in the uptake of ligands using endocytosis. Although, HS is a very versatile molecule; little information is available regarding the HS-epitope role in driving efficient trafficking of virus-cargo in endosomes, trapping cell signaling molecules which otherwise may diffuse, and controlling potential virus gene expression. Understanding the type of HS that is involved in loading and unloading virus cargo, the type of HS that effects cell-to-cell communication, and secondary messengers are emerging and fascinating science, which has tremendous treatment potential. For instance, the delivery of therapeutic agents such as HS recognizing particles conjugated with anti-HS/anti-3*O*S HS peptides in nanosomes may be given to the patient. Phage display based on anti-sulfated HS peptides will be useful in this regard as well to improve therapeutic, diagnostic options, and patient outcomes. Phage display library screening is now widely used as an antiviral approach to identify ligands that bind to virus glycoproteins or cellular receptors to reduce or block virus infection. It is tempting to speculate that designing peptides which delay or interfere with virus internalization, trafficking, or that potentially stop cell communication, could be very useful on various levels.

## Heparan sulfate- a key component in actin cytoskeleton and inflammation

Cell surface HSPGs are known to influence the cell behavior and cytoskeletal organization via interaction with the numerous ligands (Martinho et al., 1996). It has been recently discovered that intercellular bridges or cytoplasmic bridges between cells expressing F-actin and HS/3-*O*S HS are indicators of cell-based oxidative stress, virus infection inflammation and cancer (Onfelt et al., 2004; Rustom et al., 2004; Vidulescu et al., 2004; Sowinski et al., 2008). Similarly, long bridges of tunneling nanotubes (TNTs) connecting two or more cells that express HS and 3-*O*S HS (Rustom et al., 2004; Chang et al., 2016) have been proposed to be heavily involved in organelles and vesicle transport as a part of cell to cell communication. Their usage in viral transport has gained momentum for retroviruses (Sowinski et al., 2008; Omsland et al., 2018). For instance, it was recently shown that HIV infection in the primary human macrophages results in an increased number of TNTs, which further gives the virus an opportunity to spread via a novel mechanism (Hashimoto et al., 2016). A similar mechanism may exist for multiple viruses. It is obvious that viruses use TNTs smartly and strategically to cover long distances quickly and efficiently while escaping the host immune response via compartmentalization. Again, HS may play a bigger role in the virus trafficking across TNTs by clumping or aggregating around the actin bundles followed by organizing the molecular machinery to interact with myosin binding ATPases to trigger the virus movement. HSV being a neurotropic virus may find similar TNTs between two neurons. Recently it has been shown that pathogenic α-synuclein fibrils, responsible for Parkinson disease, travel between neurons in culture inside lysosomal vesicles through tunneling nanotubes (TNTs)- a recently discovered mechanism of intercellular communication (Gallegos et al., 2015). HSV with the similar opportunity may exploit TNTs to move and find the new neighborhood to cause persistence, neuroinflammation, and potentially CNS associated neurodegenerative diseases. In regard to human metapneumovirus (HMPV), a cellular extension-based model recently has been proposed to be utilized by the virus. Interestingly, the virus has two modes of infection: cell-free infection, which is blocked by neutralizing antibodies and requires binding to HS moieties, and direct cell-to-cell infection, which is HS independent (El Najjar et al., 2016).

During inflammation, chemokines stimulate the induction of endothelial filopodia and microvilli structures, which have high levels of HS/2-*O*/6-*O*/3-*O* sulfated HS (Sowinski et al., 2008). This signifies the role of sulfated-HS in helping in the sequestration or clustering of chemokines, their gradient and presentation of the chemokine to leukocytes, and further helping in leukocytes trans-endothelial migration. Interestingly, multiple studies have shown that viruses during cell entry often reorganize their actin cytoskeleton and produce filopodia (Chang et al., 2016). Although these structures have been proposed to aid in virus infections such as surfing and trafficking, it remains to be determined if such structures further participate in the recruitment of inflammatory cells and tissue invasion. On the one hand, a study by Whittall et al. (2013) clearly demonstrated co-localization of 2-*O*, 3-*O*, and 6-*O* heparan sulfate with chemokine (CXL8) on filopodial surfaces. On the other hand, the antagonizing chemotactic activity of pro-inflammatory cytokines and angiogenic activity by the usage of HS-mimetics are proving to be very useful (Mohamed and Coombe, 2017). Therefore, such small molecules can be valuable candidates in preventing not only HSV entry but also in recruiting inflammatory cells in response to HSV-1 in ocular cells and tissues (Gangji et al., 2018). This will bring a much-needed benefit for the patient suffering either from high tittered acute infection or low dosage chronic immune-mediated response to infection. The later phase is responsible for substantial immune cell infiltrates leading to scarring of the eye tissues.

## Heparan sulfate structures that interact with viral glycoproteins

Although biopolymer HS is massively diverse, specific sequences within it are likely to be critical for recognition of viral glycoproteins. An example of specific recognition is demonstrated by the HS–gD interaction, wherein a 3-*O*-sulfated GlcNp residue is required for HSV-1 to penetrate cells (Shukla et al., 1999; Xia et al., 2002; Tiwari et al., 2004). The site in HS to which gD binds is generated by an isoform of 3-*O*-sulfotransferase that yields an octasaccharide, ΔUA-(1➔4)-GlcNp2S-(1➔4)-IdoAp2S-(1➔4)-GlcNp2Ac-(1➔4)-GlcAp2S (or IdoAp2S)-(1➔4)-GlcNp2S-(1➔4)-IdoAp2S-(1➔4)-GlcNp3S6S (Liu et al., 2002). Although gB and gC are major components of the viral envelope that facilitates binding to HS, the structure(s) in HS which these glycoproteins recognize have not yet been determined. However, it appears likely that these glycoproteins recognize different HS sequence(s) as suggested by competition studies (Herold et al., 1995). In addition, different HS structures appear to be recognized by gC from HSV-1 and HSV-2 (Gerber et al., 1995). This raises the concept of HS mimetics as inhibitors of viral infection of host cells.

### Mimics of HS as inhibitors of viral entry into cells

Decades ago in 1964, it was discovered that heparin, a glycosaminoglycan related to heparan sulfate, inhibited HSV infection, which was possibly the origin for the idea that heparan sulfate is a viral receptor. Inhibition by heparin is a competitive process and, therefore, is most effective when the inhibitor is present during the attachment phase of viral entry. Heparin has been shown to bind to soluble forms of gB, gC, and gD (Nahmias and Kibrick, 1964; Herold et al., 1995; Tal-Singer et al., 1995; Feyzi et al., 1997; Trybala et al., 2000). Interestingly, whereas 6-*O-* and 3-*O*-sulfation is the primary determinant for HSV-1 infection, they appear to have a little role in HSV-2 infection, suggesting differences between the two types of viruses (Herold et al., 1996). Likewise, *O*-sulfation was found to be more important for binding to gB from HSV-1 than gB from HSV-2. Fractionation of polymeric heparin into discrete sulfated oligosaccharide mixtures suggests that nearly 10–12 monosaccharides are necessary for effective binding to gC (Feyzi et al., 1997).

Numerous other sulfated polysaccharides have been explored as mimics of HS in the inhibition of herpesvirus entry into cells. A principal source of these sulfated polysaccharides is sea algae, which biosynthesizes these molecules to retain K^+^ and Ca^+2^ ions from seawater and for enhanced resistance to desiccation. These polysaccharides include κ and λ carrageenans (Marchetti et al., 1995; Carlucci et al., 1997, 1999a,b; Zacharopoulos and Phillips, 1997), galactan sulfate (Damonte et al., 1996; Mazumder et al., 2002), galactofucan sulfate (Thompson and Dragar, 2004), fucan sulfate (Preeprame et al., 2001), spirulan (Hayashi K. et al., 1996; Hayashi T. et al., 1996), fucoidan (Ponce et al., 2003; Lee et al., 2004a), rhamnan sulfate (Lee et al., 1999), chitin sulfate (Ishihara et al., 1993), and other uncharacterized polymers (Hasui et al., 1995; Witvrouw and De Clercq, 1997; Lee et al., 2004b; Zhu et al., 2004). In addition, other polysaccharides investigated as mimics of heparan sulfate include chemically modified heparins (Herold et al., 1995, 1997; Feyzi et al., 1997), non-anticoagulated heparin (Herold et al., 1997), pentosan polysulfate (Herold et al., 1997), and dextran sulfate (Marchetti et al., 1995; Neyts et al., 1995; Dyer et al., 1997; Herold et al., 1997).

### Non-saccharide mimetics of HS as inhibitors of viral entry into cells

A growing a class of small molecules these days is called non-saccharide glycosaminoglycan (GAG) mimetics (NSGMs). NSGMs are much smaller than polymeric HS mimetics, which makes them more drug-like in nature in comparison to the oligomeric and polymeric HS mimetics. NSGMs are also much easier to prepare (synthesize) in comparison to oligomeric HS mimetics and they are highly water soluble. Most importantly, NSGMs are functional mimetics of polymeric GAGs, which arises from their ability to bind to sites on proteins that interact with GAGs. Many NSGMs have been discovered so far that modulate various processes in addition to viral infection (Desai, 2013). For example, sulfated flavonoids and xanthones have been found to work as anticoagulant and antiplatelet agents (Al-Horani et al., 2011; Correia-da-Silva et al., 2011); sulfated benzofurans have been found as inhibitors of thrombin (Sidhu et al., 2013); and sulfated benzylated glycosides have been discovered as inhibitors of human factor Xia (Al-Horani et al., 2013; Al-Horani and Desai, 2014).

In a recent work, Gangji et al. (2018) show for the first time NSGMs present an excellent alternative to polymeric HS agents for inhibiting HSV (Gangji et al., 2018). These authors screened a small library of synthetic NSGMs (MW in range of 500–2500) and identified a distinct group of NSGMs that bind glycoprotein D with high affinity (10 nM or so). More importantly, one specific NSGM, called SPGG, inhibited cellular entry of HSV-1 with *IC*_50_ in the range of 430 nM to 1.0 μM. This is greater than a 10-fold lower response than that reported for 3-*O*-sulfate containing heparin/heparan sulfate-derived octasaccharides (Liu et al., 2002; Copeland et al., 2008; Hu et al., 2011).

Overall, competitive inhibition of viral entry through the use of either oligosaccharidic or non-saccharidic agents is very exciting. Several poly/oligosaccharides and small aromatic agents have been discovered that present major opportunities for development of anti-virals. It appears that the category of NSGMs represents excellent agents for targeting viral glycoproteins.

## Epigenetic components

**Figure 4:**
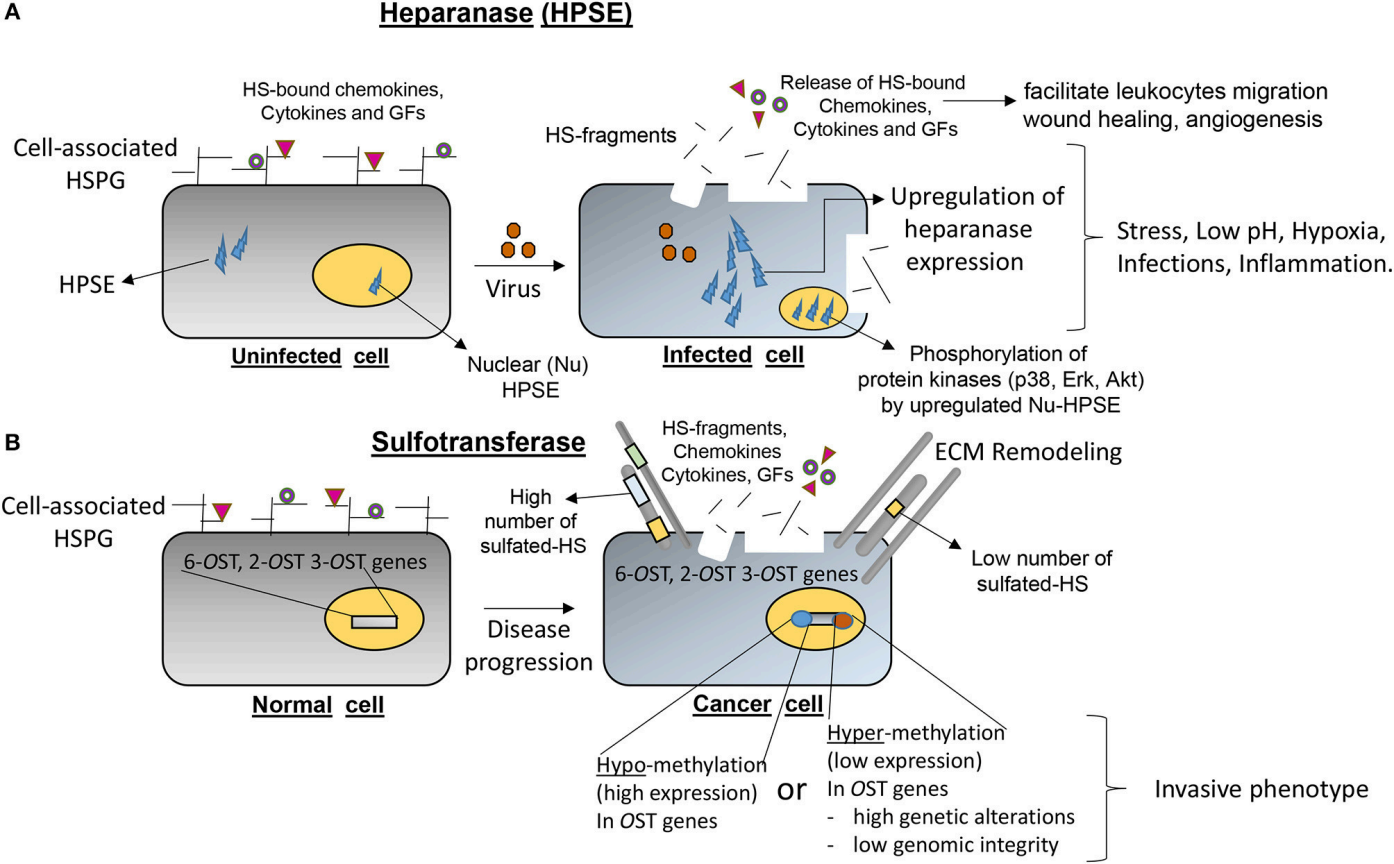
Emerging roles of heparanase (HPSE) and heparan sulfate (HS) modifying O-sulfotransferases (OSTs) in contributing directly in the onset of several disease pathologies. **(A)** An upregulation of HPSE under stress conditions such as low-pH, hypoxia, microbial infections, and inflammation degrades the normal architecture and basement membranes resulting the shedding of HSPG, cytokines/chemokines/growth factors (GFs) in ECM to initiate the cascade of inflammatory process. **(B)** Epigenetic regulations (hypo-methylation or hyper-methylation) of HS-modifying enzymes (2-, 6-, and 3-*O*STs) resulting in either lower or over-expression of *O*ST genes are widely documented in multiple diseases including cancer progression and certain invasive cancer phenotypes.

Equally important, growing evidence suggests that epigenetic regulation of HS-modifying enzymes (sulfotransferases) together with heparanase (HPSE; mammalian endoglycosidase which degrades HSPG) are important determinants in the pathogenesis of several inflammatory conditions (Figure 4). For example, Bui et al. (2010) showed upon analysis of chondrosarcoma cells the typical hypermethylation profile of 3-*O*ST sulfotransferase genes, which contributed toward the invasive phenotype of cancer. A similar hypermethylation pattern in 3-*O*ST-2 genes has recently been reported in a variety of cancers (Hwang et al., 2013; Hull et al., 2017). Several studies have shown that DNA methylation of the HPSE promoter influences HPSE expression in different stages of breast cancer and has a direct effect on tumor progression (Jiao et al., 2014). The knocking down and overexpression experiments with HPSE confirmed that HPSE regulates the transcription of a distinct cohort of immune response genes involved in T cell effector function and migration (Parish et al., 2013). An increase in HPSE levels result in NF-kB activation followed by the release of tumor-promoting substances, growth factors (GFs) and cytokines by tumor-associated macrophages (Goldberg et al., 2013). Further, the increased metastatic potential *in vivo* mice was inhibited with laminaran sulfate, a potent inhibitor of HPSE activity (Shteper et al., 2003). In addition, HPSE promotes cancer and related inflammatory pathologies by removing extracellular barriers for serve to limit invasion/extravasation. The release of HS-bound GFs and cytokines results in activation of anti-apoptotic signaling and stimulates angiogenesis (Goldberg et al., 2013). During ocular HSV-1 infection, it has been shown that upregulation of HPSE at the nucleus caused decreased interferon signaling and increased NF-κB activation, resulting neighboring cells to be more susceptible to infection and increased pro-inflammatory factor production (Agelidis et al., 2017). Therefore, HPSE represents an attractive target for the development of broad spectrum drugs which may have antimicrobial, anti-inflammatory to anti-tumor activities (Khachigian and Parish, 2004). Antimicrobial peptides and HS-mimetic, which target HPSE, are already gaining wide popularity to resolve life-threatening situations (Martin et al., 2015; Brennan et al., 2016).

The ultimate benefit of understanding the regulation of the sulfotransferase and HPSE genes and their localization mechanisms during disease development will be instrumental in the discovery of novel biomarkers. This approach will be beneficial for the early detection of disease and therefore may offer a better prognosis. In addition, targeting genes responsible for abnormal phenotype by developing highly specific inhibitors may have limited side effects and hence bring novel interventions to prevent disease.

## Conclusion

Growing evidence suggests that the interaction between viral components and their preferred modified HS-type variants is significantly important in our understanding toward the virus pathogenicity. Scientists of the past decade have developed sophisticated information on the molecular determinants of modified-HS and their counterpart ligands involved in various steps of microbial pathogenesis. Nonetheless, more exceedingly advanced works are required to demonstrate precise and more specialized work on the role of the modified forms of HS-variants in viral pathogenesis and in disease development. Heparan sulfate (HS) once stood for an ancient yet vital regulator of cell homeostasis, however with the discovery of the HS modifying enzymes, multiple roles of HS-variants (i.e., modified versions of HS) are turning out to be equally fascinating since the domains in the sulfated-HS carry very precise functions. One question remains to be addressed; do the pathogens including viruses have the ability to directly or indirectly control the expression of HS modifying enzymes? Further, the structure-function analysis of HS-variants in terms of their role in virus-infectivity, tropism, and in the disease-phenotype remains to be investigated. Since viral glycoproteins have unique structural motifs to bind HS and 3-*O*S HS, the possibility now exists to design HS mimetics, which selectively block viral protein interaction with the host cell to expand the selective therapeutic potential of HS mimetics (Gangji et al., 2018). The fact that HS, modified HS, together with HPSE activity plays a critical role during viral infection suggests that cell surface proteoglycans are a promising area for the development of reagents and understanding of the disease process. Overall, these studies certainly are worth pursuing to advance our approach to understanding disease and developing novel therapeutic interventions.

## Author contributions

DDK, DJ, MH, JB, MVV, and VT wrote the article. URD provided HS-mimetic section. VT, MH, JCB, and DDK prepared the figures and the table.

### Conflict of interest statement

The authors declare that the research was conducted in the absence of any commercial or financial relationships that could be construed as a potential conflict of interest.
